# Reduced caveolae density in arteries of SHR contributes to endothelial dysfunction and ROS production

**DOI:** 10.1038/s41598-019-43193-8

**Published:** 2019-04-30

**Authors:** Simone R. Potje, Marcella D. Grando, Andreia Z. Chignalia, Cristina Antoniali, Lusiane M. Bendhack

**Affiliations:** 1 Department of Physics and Chemistry, Faculty of Pharmaceutical Sciences of Ribeirão Preto, University of São Paulo, Ribeirão Preto, São Paulo Brazil; 2 Department of Anesthesiology, College of Medicine, University of Arizona, Tucson, Arizona United States; 3 Department of Basic Sciences, School of Dentistry, State University of São Paulo, Araçatuba, São Paulo Brazil

**Keywords:** Hypertension, Electron microscopy

## Abstract

Caveolae are plasma membrane invaginations enriched with high cholesterol and sphingolipid content; they also contain caveolin proteins in their structure. Endothelial nitric oxide synthase (eNOS), an enzyme that synthesizes nitric oxide (NO) by converting L-arginine to L-citrulline, is highly concentrated in plasma membrane caveolae. Hypertension is associated with decreased NO production and impaired endothelium-dependent relaxation. Understanding the molecular mechanisms that follow hypertension is important. For this study, we hypothesized that spontaneously hypertensive rat (SHR) vessels should have a smaller number of caveolae, and that the caveolae structure should be disrupted in these vessels. This should impair the eNOS function and diminish NO bioavailability. Therefore, we aimed to investigate caveolae integrity and density in SHR aortas and mesenteric arteries and the role played by caveolae in endothelium-dependent relaxation. We have been able to show the presence of caveolae-like structures in SHR aortas and mesenteric arteries. Increased phenylephrine-induced contractile response after treatment with dextrin was related to lower NO release. In addition, impaired acetylcholine-induced endothelium-dependent relaxation could be related to decreased caveolae density in SHR vessels. The most important finding of this study was that cholesterol depletion with dextrin induced eNOS phosphorylation at Serine^1177^ (Ser^1177^) and boosted reactive oxygen species (ROS) production in normotensive rat and SHR vessels, which suggested eNOS uncoupling. Dextrin plus L-NAME or BH_4_ decreased ROS production in aorta and mesenteric arteries supernatant’s of both SHR and normotensive groups. Human umbilical vein endothelial cells (HUVECs) treated with dextrin confirmed eNOS uncoupling, as verified by the reduced eNOS dimer/monomer ratio. BH_4_, L-arginine, or BH_4_ plus L-arginine inhibited eNOS monomerization. All these results showed that caveolae structure and integrity are essential for endothelium-dependent relaxation. Additionally, a smaller number of caveolae is associated with hypertension. Finally, caveolae disruption promotes eNOS uncoupling in normotensive and hypertensive rat vessels and in HUVECs.

## Introduction

Caveolae are flask-shaped plasma membrane invaginations enriched with cholesterol and fatty acids bearing straight, saturated chains, which make the structure rigid and highly organized^1^. Caveolae exist in many types of cells such as smooth muscle cells and adipocytes, but they are extremely abundant in endothelial cells^2,3^.

Caveolin-1 (Cav-1), a protein of 21 to 22 kD, is essential to keep caveolae organized. In agreement with this observation, lymphocytes and epithelial cells lacking caveolae and transfected with VIP21-Cav-1 cDNA induce caveolae formation^4,5^. In addition, Cav-1 knockout mice do not present caveolae in Cav-1-expressing tissues^6,7^, and Cav-1/Cav-3 double knockout mice do not contain caveolae in endothelial cells, smooth muscle cells, adipocytes, skeletal muscle fibers, or cardiac myocytes^8^. Therefore, Cav-1 is an excellent biochemical marker for caveolae formation and stabilization. Moreover, Cav-1 acts as chaperone and scaffold domain and may interact with various proteins to regulate signal transduction in caveolae^9^.

Endothelial nitric oxide synthase (eNOS) is a particularly interesting protein that remains associated with Cav-1^10^. The enzyme eNOS generates nitric oxide (NO) by converting L-arginine to L-citrulline, a process that is regulated by intracellular calcium concentration and phosphorylation mediated by upstream kinases^11^. The absence of caveolae impairs eNOS activity^12^, NO production, calcium signaling in the cardiovascular system, and endothelium-dependent relaxation^13–17^. Thus, caveolae disruption may be a factor underlying cardiovascular diseases, including essential hypertension.

Spontaneously hypertensive rat (SHR) is an animal model that is used to study the established human essential hypertension^18^. Just as in humans, hypertension develops progressively in SHRs. Furthermore, hypertension progresses through different stages, namely the pre-hypertensive, gradual, and sustained hypertensive phases^19^, worsens with aging, and becomes more severe in males than in females^20^. SHR and normotensive Wistar-Kyoto rats (WKY) show similar aortic NOS activity^21^, but eNOS expression is higher in SHR aortas as compared to WKY aortas^21–23^. In contrast, NOS activity is lower in SHR mesenteric arteries as compared to WKY mesenteric arteries. However, SHR and WKY mesenteric arteries have similar eNOS expression^21^. Additionally, compared to WKY aortas, SHR aortas have down-regulated Cav-1 expression^22,23^ and exhibit decreased NO production and impaired endothelium-dependent relaxation^24,25^. Another model of hypertension, renal hypertensive rats (2 Kidney-1 Clip, 2K-1C), has a smaller number of caveolae in aorta endothelial cells, which is related to impaired acetylcholine-induced relaxation effect^26^. Nevertheless, there are no data regarding the caveolae role and density in SHR aortas and mesenteric arteries.

Understanding the molecular mechanisms following hypertension is important. For this study, we hypothesized that SHR vessels should have a smaller number of caveolae, and that the caveolae structure should be disrupted in these vessels. This should impair the eNOS function and diminish NO bioavailability. Therefore, we aimed to investigate caveolae integrity and density in SHR aortas and mesenteric arteries, the role caveolae play in endothelium-dependent relaxation, and the mechanisms ensuing after caveolae disruption. We compared data obtained with SHR and Wistar rat vessels.

## Results

### Caveolae disruption reduced acetylcholine (ACh)-induced endothelium-dependent relaxation

**Figure 1:**
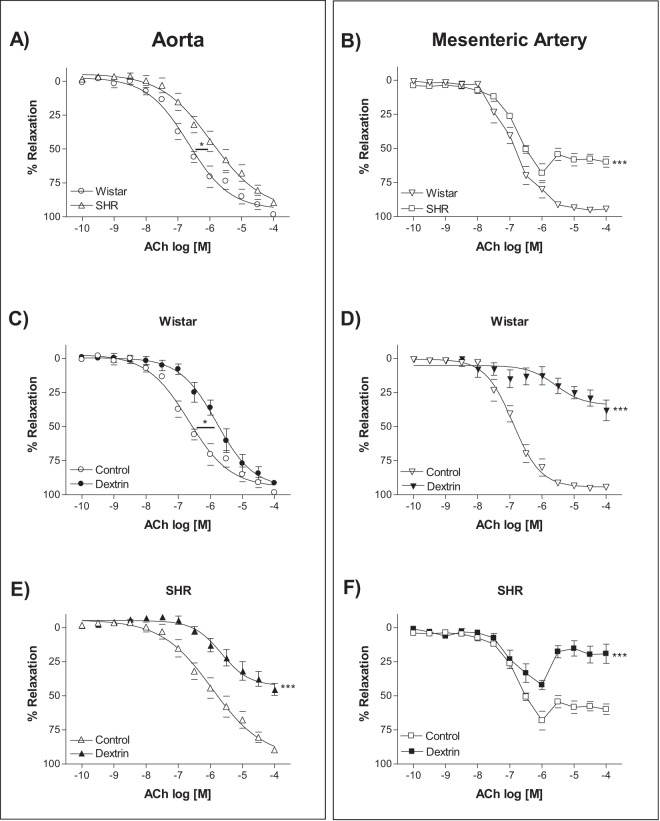
Concentration-response curves constructed for the effect of acetylcholine (ACh, 0.1 nM to 0.1 mM) on normotensive rat and SHR (n = 5–7) endothelium-intact aortas (**A**,**C**,**E**) and mesenteric arteries (**B**,**D**,**F**) in the absence (Control) or in the presence of methyl-β-cyclodextrin (10 mM dextrin, for 60 min). Data represent the mean ± SEM of the experiments, and *n* represents the number of aortic or mesenteric rings used in the experiments. *p < 0.05 statistical difference in pD_2_ values between SHRs *versus* normotensive rats and Dextrin *versus* Control groups. ***p < 0.001 statistical difference in maximum relaxant effect values between SHRs *versus* normotensive rats and Dextrin *versus* Control groups.

ACh-induced endothelium-dependent relaxation decreased in the SHR aortas (Fig. 1A) and mesenteric arteries (Fig. 1B). In the aortas, stimulation with ACh elicited similar maximum relaxant effects in normotensive rats (98.4 ± 0.3%, n = 5) and SHRs (89.6 ± 0.6%, n = 7), but the ACh potency was lower in the SHR aortic rings (pD_2_: 5.75 ± 0.4, n = 7) than in the normotensive rat aortic rings (pD_2_: 6.51 ± 0.1, n = 5). On the other hand, the ACh potency values were similar in the normotensive rat (pD_2_: 6.94 ± 0.1, n = 7) and the SHR (pD_2_: 6.92 ± 0.05, n = 6) mesenteric arteries, whereas the ACh-stimulated maximum relaxant effect was impaired in SHRs (60.0 ± 3.9%, n = 6) as compared to normotensive rats (94.5 ± 1.7%, n = 7).

To analyze the caveolae contribution to the ACh-induced endothelium-dependent relaxation, we used dextrin, an agent that depletes membrane cholesterol that is essential for caveolae stability^27^. In the normotensive rat aortas, dextrin not changed the ACh-stimulated maximum relaxant effect (91.4 ± 2.0%, n = 7), but it reduced the ACh potency (pD_2_: 5.72 ± 0.1, n = 7) (Fig. 1C). In the SHR aortas, dextrin impaired the ACh-induced maximum relaxant effect (45.2 ± 4.4%, n = 7) (Fig. 1E). Moreover, dextrin impaired the ACh-induced maximum relaxant effect in the normotensive rat (38.0 ± 7.5%, n = 6) (Fig. 1D) and SHR (19.1 ± 7.0%, n = 6) (Fig. 1F) mesenteric arteries.

### Caveolae disassembly increased phenylephrine (PE)-induced contraction

**Figure 2:**
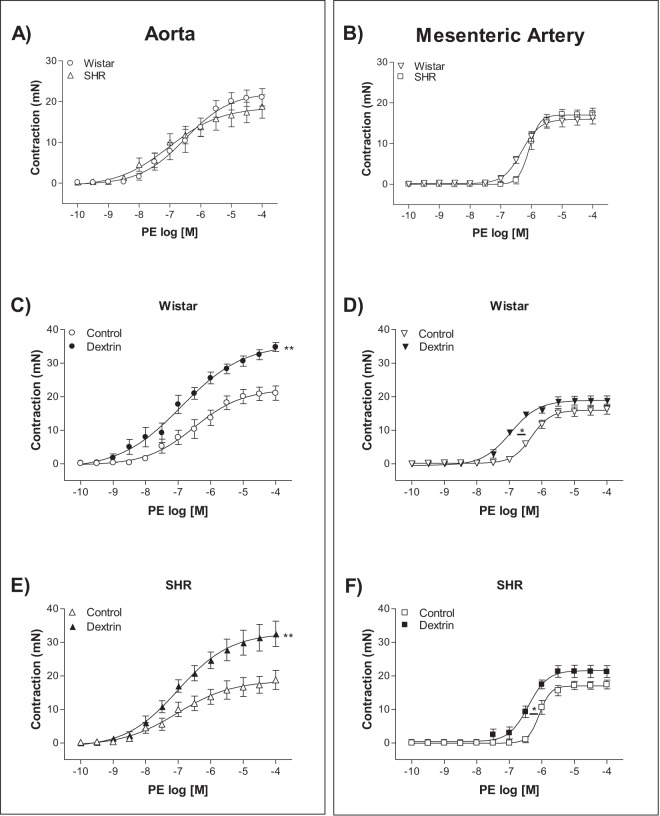
Concentration-response curves constructed for the effect of phenylephrine (PE, 0.1 nM to 0.1 mM) on normotensive rat and SHR (n = 5–8) endothelium-intact aortas (**A**,**C**,**E**) and mesenteric arteries (**B**,**D**,**F**) in the absence (Control) or in the presence of methyl-β-cyclodextrin (10 mM dextrin, for 60 min). Data represent the mean ± SEM of the experiments, and *n* represents the number of aortic or mesenteric artery rings used in the experiments. *p < 0.05 statistical difference in pD_2_ values between Dextrin *versus* Control groups. **p < 0.01 statistical difference in maximum contractile effect values between Dextrin *versus* Control groups.

PE induced similar contractile responses in the normotensive rat and SHR aortas and mesenteric arteries (Fig. 2A,B). Dextrin increased the PE-induced maximum contractile effect in the normotensive rat and SHR aortas (Fig. 2C,E). On the other hand, dextrin did not alter the PE-induced maximum contractile effect in the normotensive rat or SHR mesenteric arteries, but it increased the PE potency (Fig. 2D,F). Table 1 lists the maximum contractile effect and the pD_2_ values of the concentration-response curves constructed for the normotensive rat and SHR aortic and mesenteric artery rings in the presence of PE.

**Table 1:** Maximum contractile effect and pD_2_ (Negative logarithm of the EC_50_, concentration of the agent that produced half-maximal amplitude) induced by phenylephrine in aortas and mesenteric arteries of Wistar and SHR, treated (dextrin) or not (control) with dextrin (10 mM for 60 min).

	Phenylephrine			
	Wistar		SHR	
	Aorta (Control)	Aorta (Dextrin)	Aorta (Control)	Aorta (Dextrin)
Maximum contractile effect (mN)	21.0 ± 2.1, n = 6	34.7 ± 1.3**, n = 8**	18.7 ± 2.8, n = 7	32.5 ± 3.7**, n = 7
pD_2_	6.5 ± 0.2, n = 6	6.8 ± 0.4, n = 8	7.0 ± 0.2, n = 7	7.3 ± 0.2, n = 7
	Mesenteric artery (Control)	Mesenteric artery (Dextrin)	Mesenteric artery (Control)	Mesenteric artery (Dextrin)
Maximum contractile effect (mN)	16.2 ± 1.4, n = 6	18.8 ± 1.4, n = 5	17.4 ± 1.3, n = 6	21.2 ± 1.7, n = 7
pD_2_	6.3 ± 0.1, n = 6	6.9 ± 0.1*, n = 5	6.0 ± 0.1, n = 6	6.4 ± 0.1*, n = 7

*p < 0.05 and **p < 0.01 between Dextrin *versus* Control groups.

### The number of caveolae decreased during hypertension

**Figure 3:**
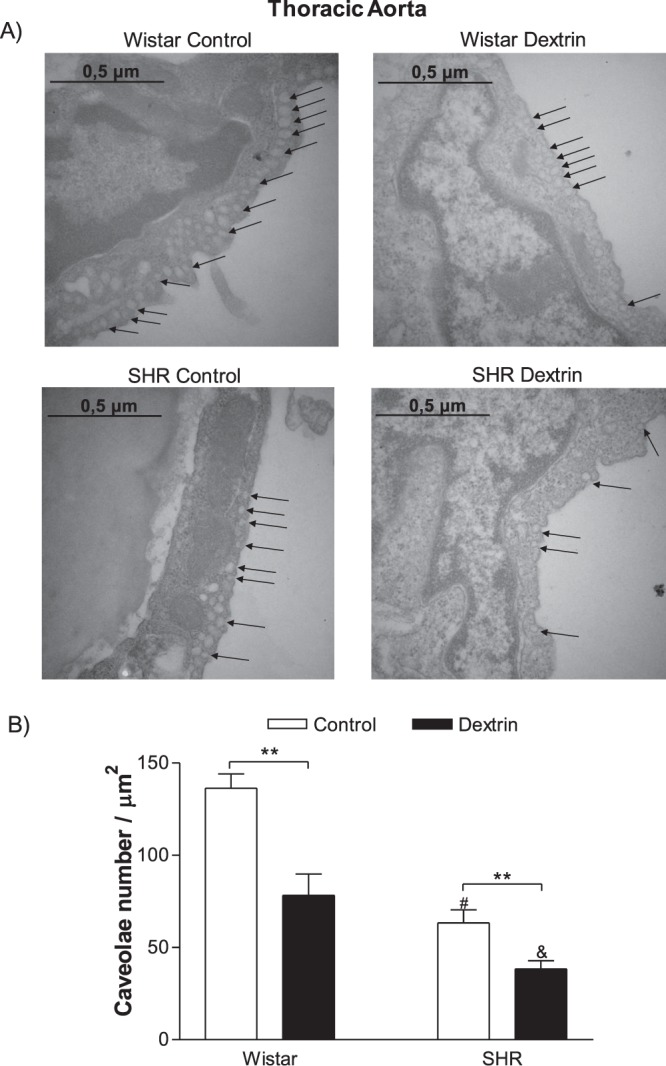
(**A**) Identification of caveolae-like structures on the surface of intact endothelial cells of normotensive rats and SHR aortas by transmission electron microscopy. General arrangement representative of the control groups on the left side and of the groups treated with methyl-β-cyclodextrin (10 mM dextrin, for 60 min) on the right side. Original magnification of 50,000× and 0.5-μm scale bar in each case. (**B**) Number of caveolae/μm^2^ in aortic endothelial cells from normotensive rats and SHRs, treated with methyl-β-cyclodextrin (dextrin) or not (control). Bars represent the mean ± SEM of five electron micrographs counted in areas of similar magnitudes. The values are expressed as the number of caveolae per area (μm^2^). **p < 0.01 statistical difference in caveolae density values between Dextrin *versus* Control groups in both normotensive rats and SHRs; ^#^p < 0.05 statistical difference in caveolae density in the control groups between SHRs *versus* normotensive rats; ^&^p < 0.05 statistical difference in caveolae density in SHRs treated with dextrin *versus* other groups.

**Figure 4:**
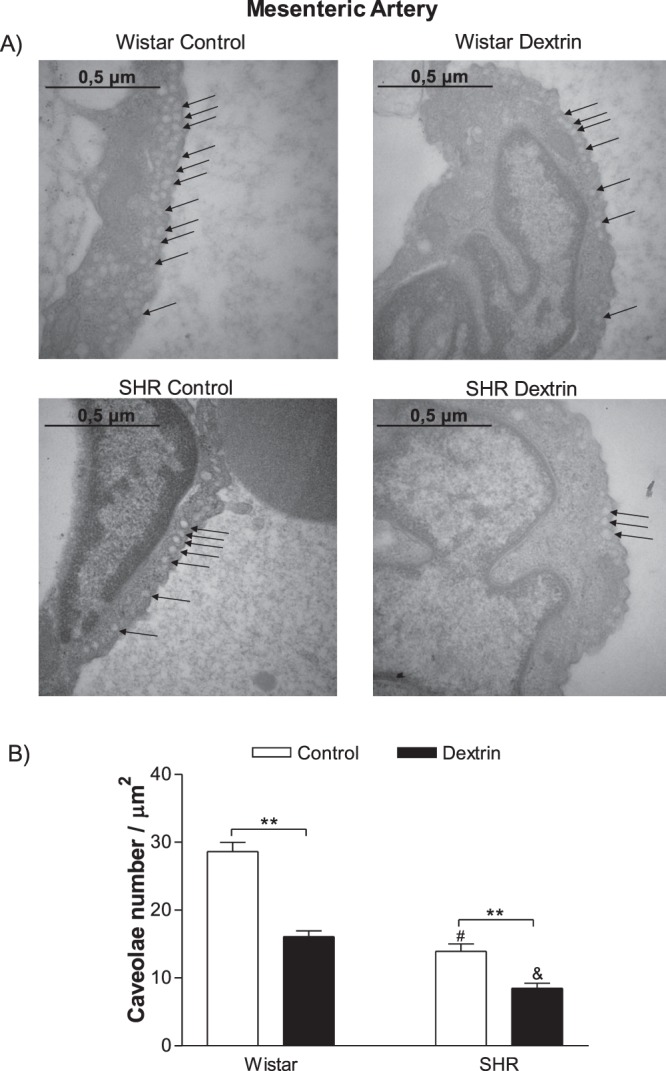
(**A**) Representative electron micrographs of caveolae-like structures in normotensive rat and SHR mesenteric arteries treated with methyl-β-cyclodextrin (10 mM dextrin, for 10 min, right side) or not (control, left side). Original magnification of 50,000× and 0.5-μm scale bar in each case. (**B**) Number of caveolae/μm^2^ in mesenteric artery endothelial cells from normotensive rats and SHR treated with methyl-β-cyclodextrin (dextrin) or not (control). Bars represent the mean ± SEM of five electron micrographs counted in areas of similar magnitudes. The values are expressed as the number of caveolae per area (μm^2^). **p < 0.01 statistical difference in caveolae density values between Dextrin *versus* Control groups in both normotensive rats and SHRs; ^#^p < 0.05 statistical difference in caveolae density in the control groups between SHRs *versus* normotensive rats; ^&^p < 0.05 statistical difference in caveolae density in SHRs treated with dextrin *versus* other groups.

To analyze whether the number of caveolae was lower in the SHR aortas and mesenteric arteries as compared to normotensive rats and to investigate whether caveolae disruption with dextrin reduced caveolae integrity, we carried out electron transmission microscopy experiments. Figures 3B and 4B show that the normotensive rat vessels contained a larger number of caveolae (aortas: 136 ± 7 caveolae/μm^2^, n = 5; mesenteric arteries: 28 ± 1 caveolae/μm^2^, n = 5) as compared to the SHR vessels (aortas: 63 ± 7 caveolae/μm^2^, n = 5; mesenteric arteries: 13 ± 1 caveolae/μm^2^, n = 5). In both normotensive rats and SHRs, dextrin reduced the number of caveolae in the aortas (normotensive rats: 78 ± 11 caveolae/μm^2^, n = 5; SHRs: 38 ± 4 caveolae/μm^2^, n = 5) and mesenteric arteries (normotensive rats: 16 ± 1 caveolae/μm^2^, n = 5; SHRs: 8 ± 1 caveolae/μm^2^, n = 5). Figures 3A and 4A respectively depict the representative electron micrographs of caveolae-like structures in the normotensive rat and SHR aortas and mesenteric arteries treated with dextrin (10 mM) for 60 min or not (control).

### Caveolae structural disruption promoted eNOS phosphorylation

**Figure 5:**
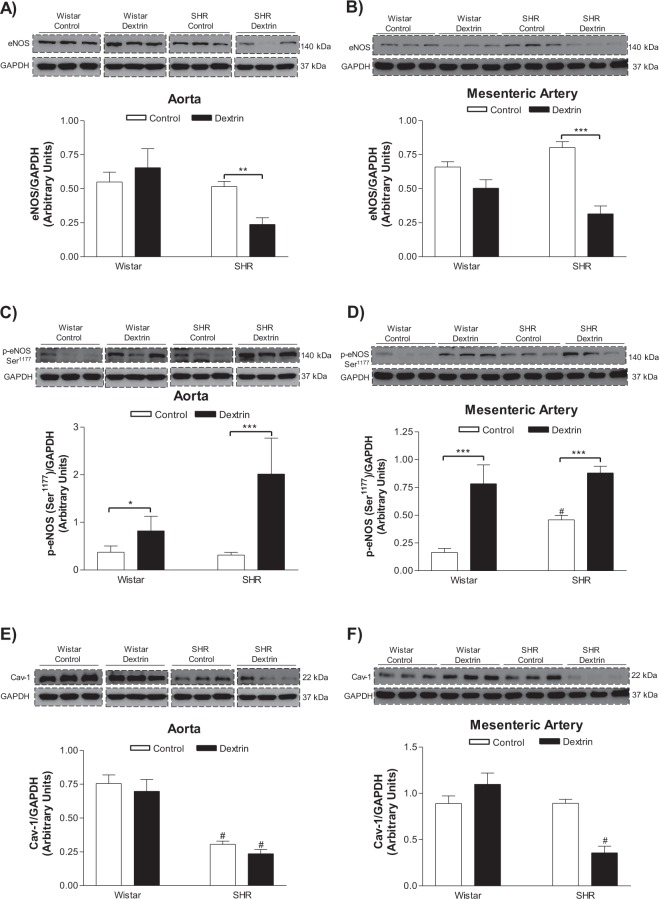
Representative western blot of total eNOS (top blot), eNOS phosphorylation at Serine^1177^ (top blot), caveolin-1(top blot), and GAPDH (bottom blot; loading control) in normotensive rat and SHR aortas (**A**,**C**,**E**) and mesenteric arteries (**B**,**D**,**F**) treated with methyl-β-cyclodextrin (10 mM dextrin, for 60 min) or not (Control). We processed images of blots changing brightness and contrast and we applied equally over the entire image and controls. *p < 0.05, **p < 0.01, ***p < 0.001 statistical difference between Dextrin *versus* Control groups; ^#^p 0.05 statistical difference between SHR versus normotensive rat groups.

The aortas (normotensive rats: 0.54 ± 0.07, n = 6; SHRs: 0.51 ± 0.03, n = 6) and mesenteric arteries (normotensive rats: 0.65 ± 0.03, n = 6; SHRs: 0.80 ± 0.04, n = 6) of normotensive rats and SHRs had similar eNOS expression (Fig. 5A,B). Treatment with dextrin did not modify the eNOS expression in the normotensive rat vessels (Aortas: 0.65 ± 0.13, n = 6; Mesenteric arteries: 0.50 ± 0.06, n = 6), but it decreased the eNOS expression in the SHR vessels (Aortas: 0.23 ± 0.04, n = 6; Mesenteric arteries: 0.31 ± 0.05, n = 6) (Fig. 5A,B). eNOS phosphorylation at Serine^1177^ (Ser^1177^) was similar in the SHR aortas (0.31 ± 0.05, n = 6) and the normotensive rat aortas (0.37 ± 0.13, n = 6) (Fig. 5C). However, eNOS-Ser^1177^ phosphorylation was more pronounced in the SHR mesenteric arteries (0.45 ± 0.04, n = 6) as compared to the normotensive rat mesenteric arteries (0.16 ± 0.03, n = 6) (Fig. 5D). Dextrin increased eNOS-Ser^1177^ phosphorylation in the normotensive rat and SHR aortas (normotensive rats: 0.82 ± 0.30, n = 6; SHRs: 2.02 ± 0.75, n = 6) and mesenteric arteries (normotensive rats: 0.72 ± 0.17, n = 6; SHRs: 0.88 ± 0.06, n = 6) (Fig. 5C,D). Cav-1 expression was lower in the SHR aortas (0.30 ± 0.0, n = 6) as compared to the normotensive rat aortas (0.75 ± 0.06, n = 6). Dextrin did not alter Cav-1 expression in any of the groups (normotensive rat aortas: 0.69 ± 0.08, n = 6; SHR aortas: 0.23 ± 0.03, n = 6) (Fig. 5E). On the other hand, normotensive rats (0.89 ± 0.08, n = 6) and SHRs (0.89 ± 0.04, n = 6) displayed similar Cav-1 expression in the mesenteric arteries. After treatment with dextrin, though, Cav-1 expression diminished in the SHR mesenteric arteries (0.35 ± 0.07, n = 6), but not in the normotensive rat mesenteric arteries (1.09 ± 0.12, n = 6) (Fig. 5F).

### Caveolae structural disassembly reduced NO_x_ levels and increased ROS levels

**Figure 6:**
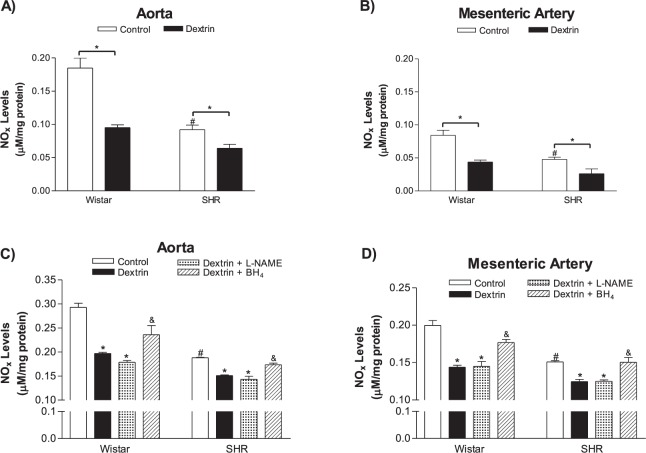
NOx levels in normotensive rat and SHR aortic (**A**,**C**) and mesenteric artery (**B**,**D**) rings treated with methyl-β-cyclodextrin (10 mM dextrin, for 60 min) or not (control) (n = 5–10). Some rings were incubated in presence of L-NAME (1 mM, 30 min) or BH_4_ (100 µM, 30 min) (**C**,**D**). Bars represent the mean ± SEM of the experiments, and *n* represents bath solutions from the different aortic or mesenteric artery rings used herein. *p < 0.05 statistical difference in NOx level values between Dextrin or Dextrin plus L-NAME *versus* Control groups; ^#^p < 0.05 statistical difference in NOx level values in control groups between SHRs *versus* normotensive rats; ^&^p < 0.05 statistical difference in NOx level between Dextrin plus BH_4_ *versus* Dextrin groups.

We detected lower NOx levels, as measured with the Griess reagent in the bath solutions of the ACh-stimulated SHR aortic rings (0.0921, n = 10) as compared to the NOx levels detected in the bath solutions of the normotensive rat aortic rings (0.1847, n = 10). Pre-treatment of the aortic rings with dextrin reduced the NOx levels in the bath solutions of the ACh-stimulated normotensive rat (0.0952, n = 10) and SHR (0.0641, n = 10) aortic rings (Fig. 6A).

We achieved similar results for the bath solutions of the ACh-stimulated normotensive rat and SHR mesenteric artery rings. The NOx levels detected in the bath solutions of the SHR mesenteric artery rings (0.0477, n = 10) were almost 50% lower than the NOx levels verified in the bath solutions of the normotensive rat mesenteric artery rings (0.0842, n = 10). Pre-treatment with dextrin diminished the NOx levels in the bath solutions of both the normotensive rat (0.0438, n = 10) and SHR (0.0261, n = 10) mesenteric artery rings (Fig. 6B).

In another set of experiments, the aortic rings treated with dextrin (Normotensives: 0.196, n = 4; SHRs: 0.151, n = 4) or dextrin plus L-NAME (Normotensives: 0.178, n = 4; SHRs: 0143, n = 4) decreased the NOx levels compared to control group (Normotensives: 0.292, n = 4; SHRs: 0.188, n = 4). However, incubation with BH_4_ (Normotensives: 0.236, n = 4; SHRs: 0.173, n = 4) increased the NOx levels in aortic rings treated with dextrin (Fig. 6C).

Similar results were observed in mesenteric arteries rings. Treatment with dextrin (Normotensives: 0.143, n = 4; SHRs: 0.124, n = 4) or dextrin plus L-NAME (Normotensives: 0.144 ± , n = 4; SHRs: 0.125, n = 4) reduced NOx levels compared to control group values (Normotensives: 0.199, n = 4; SHRs: 0.150, n = 4). Nevertheless, the NOx levels were raised in presence of BH_4_ (Normotensives: 0.176, n = 4; SHRs: 0.150, n = 4) almost at the same values of control group (Fig. 6D).

**Figure 7:**
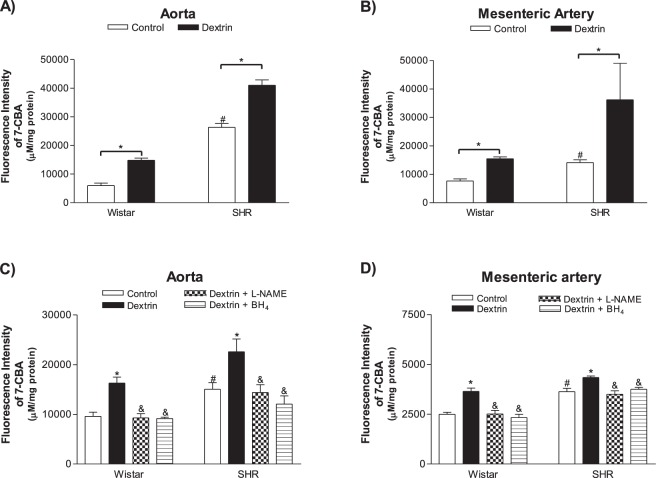
Reactive oxygen species (ROS) levels in normotensive rats and SHR aortic (**A**,**C**) and mesenteric artery (**B**,**D**) rings treated with methyl-β-cyclodextrin (10 mM dextrin for 60 min) or not (control) (n = 5), as measured with the coumarin-7-boronate acid (7-CBA) fluorescence probe. Some rings were incubated with L-NAME (1 mM, 30 min) or BH_4_ (100 µM, 30 min). Bars represent the mean ± SEM of the experiments, and *n* represents bath solutions from the different aortic or mesenteric artery rings used herein. *p < 0.05 statistical difference in ROS level values between Dextrin *versus* Control groups; ^#^p < 0.05 statistical difference in ROS level values in control groups between SHR *versus* normotensive rats; ^&^p < 0.05 statistical difference in ROS levels between Dextrin plus L-NAME or Dextrin plus BH_4_ *versus* Dextrin groups.

On the other hand, the ROS levels measured with the 7-CBA fluorescence probe were higher in the bath solutions of the ACh-stimulated SHR vessels (Aortas: 26320.0, n = 5; Mesenteric arteries: 14130.0, n = 5) than in the bath solutions of the ACh-stimulated normotensive rat vessels (Aortas: 5993.0, n = 5; Mesenteric arteries: 7672.0, n = 5) (Fig. 7A,B). After treatment with dextrin followed by stimulation of ACh, the ROS levels increased in the bath solutions of the aortas (normotensive rats: 14810.0, n = 5; SHRs: 41030.0, n = 5) and mesenteric artery rings (normotensive rats: 15460.0, n = 5; SHRs: 36249.0, n = 5) of both groups (Fig. 7A,B).

In another set of experiments, it was observed that L-NAME or BH_4_ decreased ROS production promoted by dextrin treatment compared to control groups, both in aortic rings (Fig. 7C), as well as in rings of mesenteric arteries (Fig. 7D) of normotensive and SHR (Table 2).

**Table 2:** Reactive oxygen species (ROS) production measured in aortas and mesenteric arteries of normotensive and SHR, treated (dextrin) or not (control) with dextrin (10 mM for 60 min).

	Groups	Aorta (Control)	Aorta (Dextrin)	Aorta (Dextrin + L-NAME)	Aorta (Dextrin + BH_4_)
Fluorescence intensity of 7-CBA (a.u.)	Wistar	9593 ± 831, n = 5	16280 ± 1210*, n = 5	9290 ± 829^&^, n = 5	9146 ± 275^&^, n = 5
	SHR	15030 ± 1328, n = 5	22580 ± 2562*, n = 5	14390 ± 1580^&^, n = 5	12060 ± 1649^&^,n = 5
		Mesenteric artery (Control)	Mesenteric artery (Dextrin)	Mesenteric artery (Dextrin + L-NAME)	Mesenteric artery (Dextrin + BH_4_)
Fluorescence intensity of 7-CBA (a.u.)	Wistar	2489 ± 103, n = 5	3645 ± 165*, n = 5	2507 ± 183^&^, n = 5	2330 ± 161^&^, n = 5
	SHR	3625 ± 171, n = 5	4334 ± 84*, n = 5	3496 ± 173^&^, n = 5	3747 ± 95^&^, n = 5

Some rings were incubated with L-NAME (1 mM for 30 min) or BH_4_ (100 µM for 30 min). *p < 0.05 between Dextrin *versus* Control groups. ^&^p < 0.05 between Dextrin plus L-NAME or BH_4_ *versus* Dextrin group.

### Caveolae disruption promoted eNOS uncoupling

**Figure 8:**
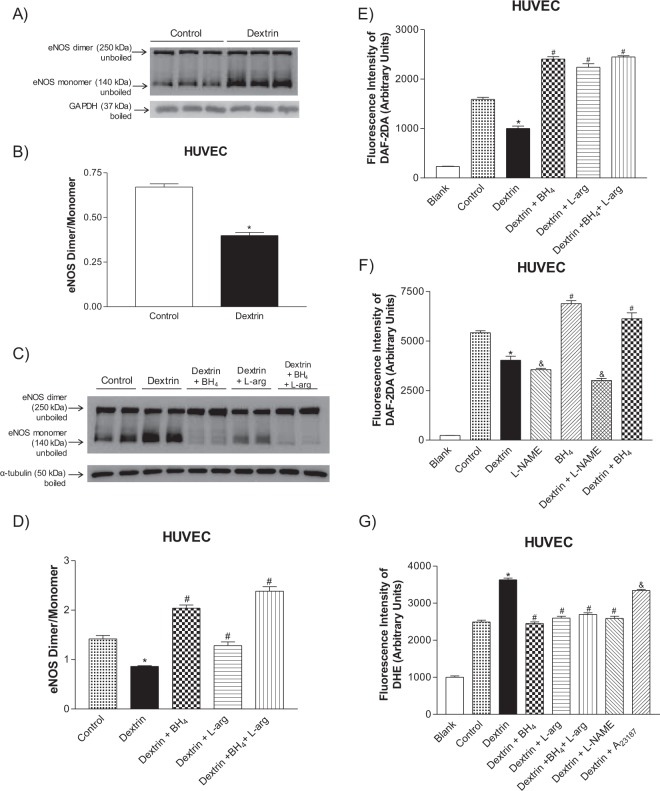
(**A**,**C**) Representative western blot of eNOS dimer/monomer distribution in unboiled samples (top blot) and GAPDH or α-tubulin (bottom blot; loading control) in human umbilical vein endothelial cells (HUVECs) treated with methyl-β-cyclodextrin (10 mM dextrin for 60 min) or not (Control). (**B**,**D**) Basal changes in eNOS dimer and monomer induced by dextrin, in the presence of tetrahydrobiopterin (BH_4_, 100 μM), L-arginine (L-arg, 1 mM), or BH_4_ plus L-arg or not. We processed images of blots changing brightness and contrast and we applied equally over the entire image and controls. (**E**,**F**) NO determination with the DAF-2DA probe in HUVECs treated with dextrin or not (Control) in the presence of BH_4_, L-arg, L-NAME (1 mM), or BH_4_ plus L-arg. (**G**) Reactive oxygen species (ROS) detected with the DHE probe in the presence of BH_4_, L-arg, BH_4_ plus L-arg, L-NAME, or A_23187_ (5 μM) in HUVECs treated with dextrin or not. Bars are reported as the mean ± SEM of independent experiments (n = 4–5). Values of blank were not use to perform statistics. *p < 0.05 statistical differences between Dextrin *versus* control group. ^#^p < 0.05 between dextrin (in the presence of BH_4_, or L-arg, or BH_4_ plus L-arg) and only BH_4_ *versus* Dextrin group. ^&^p < 0.05 between L-NAME, dextrin plus L-NAME or A_23187_ *versus* Control group.

The human umbilical vein endothelial cells (HUVECs) had lower eNOS dimer/monomer ratio after treatment with dextrin (0.39 ± 0.01 arbitrary units, n = 6) as compared to the HUVEC control (0.67 ± 0.01 arbitrary units, n = 6), which indicated eNOS uncoupling (Fig. 8A,B). To investigate whether supplementation with an eNOS co-factor or substrate would avoid the eNOS uncoupling promoted by dextrin, we used BH_4_ and L-arginine. On the basis of Fig. 8C,D, eNOS monomerization in HUVECs promoted by dextrin (0.86 ± 0.01 arbitrary units, n = 4) was prevented by treatment with BH_4_ (2.03 ± 0.06 arbitrary units, n = 4), L-arginine (1.28 ± 0.07 arbitrary units, n = 4), or BH_4_ plus L-arginine (2.23 ± 0.08 arbitrary units, n = 4) as compared to the HUVECs control (1.41 ± 0.06 arbitrary units, n = 4).

eNOS uncoupling is associated with increased ROS levels and decreased NO production. The DHE fluorescence became more intense in HUVECs in the presence of dextrin (3631 ± 46, n = 8) and dextrin plus A_23187_ (3340 ± 31, n = 8). However, pre-treatment with BH_4_ (2452 ± 41, n = 8), L-arginine (2597 ± 48, n = 8), BH_4_ plus L-arginine (2696 ± 45, n = 8), or L-NAME (2589 ± 54, n = 8) diminished the DHE fluorescence to the same level as the control group (2492 ± 49, n = 8) (Fig. 8G). On the other hand, NO measured in the HUVECs with the aid of the DAF-2DA probe decreased in the presence of dextrin (1000 ± 48, n = 8) as compared to the control (1589 ± 40, n = 8). In the presence of BH_4_ (2407 ± 47, n = 8), L-arginine (2239 ± 71, n = 8), or BH_4_ plus L-arginine (2449 ± 22, n = 8), the DAF-2DA fluorescence intensity increased above the baseline levels (Fig. 8E).

In another set of experiments, both dextrin (4030 ± 201, n = 6), L-NAME (3554 ± 70, n = 6) or dextrin plus L-NAME (3007 ± 102, n = 6) decreased DAF-2DA fluorescence intensity compared to control (5418 ± 96, n = 6). While BH_4_ (6887 ± 145, n = 6) or dextrin plus BH_4_ (6123 ± 296, n = 6) increased the fluorescence intensity of DAF-2DA above the levels of control group (Fig. 8F).

## Discussion

**Figure 9:**
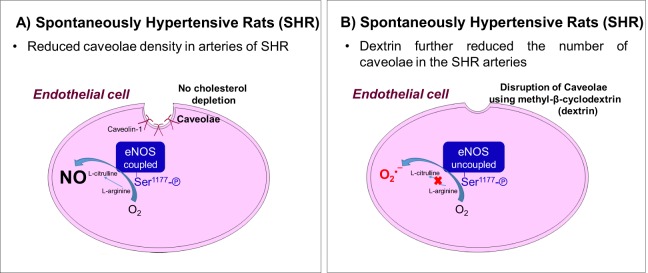
(**A**) Representative scheme of reduced caveolae density in endothelial cells of SHR arteries without cholesterol depletion. The eNOS is still able to produce NO. (**B**) In presence of methyl-β-cyclodextrin (dextrin) occurs disruption of caveolae structure promoting reduction of caveolae density and eNOS uncoupling, leading to superoxide production.

This study has shown that caveolae-like structures is decreased in SHR aortas and mesenteric arteries compared to normotensive rats. In addition, impairment of ACh-induced endothelium-dependent relaxation could be related to decreased caveolae density in SHR vessels. The most important finding of this study was that cholesterol depletion due to the presence of dextrin induced eNOS phosphorylation at Ser^1177^ and greatly increased ROS production in normotensive rat and SHR vessels, which suggested eNOS uncoupling (Fig. 9). Treatment of HUVECs with dextrin confirmed eNOS uncoupling, as evidenced by the lower eNOS dimer/monomer ratio. The presence of BH_4_, L-arginine, or BH_4_ plus L-arginine inhibited eNOS monomerization. These results showed that a reduction in the number of caveolae was associated with hypertension disease, and that caveolae disruption promoted eNOS uncoupling.

It is widely accepted that the formation of caveolae structure requires the expression of Cav-1^6,7^. A lower number of caveolae was observed in SHR vessels when compared to normotensive rat before dextrin treatment (Figs 3 and 4). Corroborating this data, Cav-1 expression was diminished in SHR aorta compared to normotensive rat (Fig. 5E). However, Cav-1 expression was similar in mesenteric arteries of SHR and normotensive rat (Fig. 5F). In an excellent review, the conditions that are necessary for caveolae structure to be formed are discussed^28^. After Cav-1 is synthetized in the endoplasmic reticulum, an oligomerization process takes place that is dependent on caveolin-2 and participation of the 8S complex on sucrose gradients^29^. This step of 8S-oligomerization appears to be essential for the proper formation of caveolae, because Cav-1 that fail to oligomerize are unable to independently turnover into caveolae^30,31^. In addition, the cavins are proteins that act as support or adapter to caveolae formation and in its absence the caveolae assembly is compromised *in vivo* in all tissues^32^. Also, cavin-1 knock-down significantly reduces caveolae number in both mammalian cells and zebrafish^33^. It is possible that other factors are not discussed here, which are required for caveolae formation are unregulated in mesenteric arteries of SHR. Further studies are required to pinpoint which step of the signaling pathway that result in caveolae formation is defective in resistance vessels of SHR.

PE-induced contraction in normotensive rat and SHR aortas and mesenteric arteries presented similar amplitude (Fig. 2A,B). After caveolae disruption, the PE-stimulated maximum contractile effect increased in normotensive rat and SHR aortas (Fig. 2C,E), and the PE potency intensified in normotensive rat and SHR mesenteric arteries (Fig. 2D,F). In contrast to our findings, a study that used male Sprague-Dawley rat aortic rings with intact endothelium reported that dextrin does not modify smooth muscle contractile responses to PE, which suggested that this α-adrenergic agonist does not require the presence of signaling molecules in cholesterol-rich domains to cause contraction^34^. Dreja and colleagues^35^ demonstrated that the α_1_-adrenergic-selective agonist cirazoline promotes similar contractions in rat-tail endothelium-denuded artery rings before and after treatment with dextrin. However, the increased PE contractile effect promoted by dextrin and verified herein could be related to release of lower basal NO levels after treatment with dextrin in both aortas and mesenteric arteries (Figs 6A and 8B). Moreover, caveolae disruption in rat femoral arteries was reported not only to reduce basal NO levels, but also to decrease the involvement of BKCa channels in smooth muscle cell membrane potential and consequently arterial contraction^36^. Furthermore, ROS levels in normotensive rat and SHR aortas and mesenteric arteries were higher after treatment with dextrin (Fig. 7A,B), and these ROS could contribute to the contraction of underlying smooth muscle cells^37^.

ACh-induced endothelium-dependent relaxation diminished in normotensive rat and SHR aortas and mesenteric arteries (Fig. 1A,B), corroborating with previous studies showing that this impairment is due to different mechanisms associated with hypertension, aging^24,38,39^ and vascular smooth muscle cells^40^. In addition, this endothelium-dependent relaxation impairment could be related to a lower number of caveolae in SHR aortas (Fig. 3) and mesenteric arteries (Fig. 4). A smaller number of caveolae was attributed to impaired ACh-induced relaxation impaired in the renal hypertensive model (2K-1C)^26^. Another study reported a greater number of caveolae in myocardium of ten-week-old SHRs (early stage of hypertension with significant cardiac hypertrophy) as compared to WKY of the same age. In 20-week-old SHRs with established hypertension and cardiac hypertrophy, the caveolae observed in 10-week-old SHRs no longer exist^41^. On the basis of these findings, caveolae play a role in the process of cardiovascular diseases, and caveolae absence or loss underlies hypertension.

eNOS is enriched in plasma membrane caveolae, and eNOS activity is nine- to ten-fold more prevalent in the caveolar fraction as compared to whole plasma membrane^42^. Normotensive rat and SHR aortas and in mesenteric arteries had similar basal eNOS expression (Fig. 5A,B). On the other hand, eNOS activity indirectly measured by the Griess assay was lower in SHR vessels as compared to normotensive rat vessels (Fig. 6A,B), which could be associated with decreased caveolae density in SHR vessels (Figs 3 and 4). After treatment with dextrin, the number of caveolae in SHR aortas and in mesenteric arteries decreased (Figs 3 and 4), so we can suggest that eNOS association with caveolae also reduced. Therefore, treatment with dextrin diminished eNOS expression just in SHR vessels (Fig. 5A,B).

Caveolae structural disruption with dextrin in normotensive rats abated ACh potency in aortas and the ACh-induced maximum relaxant effect in mesenteric arteries (Fig. 1C,D). In SHRs, dextrin reduced the ACh-stimulated maximum relaxant effect in both vessels (Fig. 1E,F). Different studies reported decreased NO release and impaired ACh-induced relaxation after caveolae disruption^14,16,26,34^, confirming that caveolae structure and integrity are essential for endothelium-dependent relaxation. Therefore, we focused on the mechanisms ensuing after caveolae disruption.

As mentioned previously, the most important finding of our study was that cholesterol depletion in caveolae due to the presence of dextrin induced eNOS phosphorylation at Ser^1177^ in normotensive rat and SHR vessels (Fig. 5C,D). This response was followed by reduced NO production (Fig. 6) and increased ROS levels (Fig. 7) in ACh-stimulated vessels obtained from normotensive rats and SHRs. Although eNOS phosphorylation at the Ser^1177^ residue regulates NO generation^43,44^, Ser^1177^ phosphorylation in uncoupled eNOS increases the ROS generation rate^45^. To show that, researchers stimulated purified BH_4_-free human endothelial NOS (heNOS) with AKT and PKCα, to observe Ser^1177^ phosphorylation in heNOS as well as 54% increase in ROS generation. However, ROS generation produced by heNOS Thr^495^ phosphorylation does not elicit any significant effects as compared to the control enzyme^45^. In order to confirm that eNOS is the source of NO and ROS production, we performed experiments in the presence of L-NAME in aortic and mesenteric rings, as well as in HUVEC. We showed that dextrin plus L-NAME did not add their effects or promoted an increased NOx levels, confirming that NO is derived from NOS (Figs 6C,D and 8F). In addition, the fluorescence intensity of 7-CBA increased by dextrin treatment was damped in presence of L-NAME or BH_4_ (Fig. 7C,D). Therefore, our results suggest that caveolae disruption promotes eNOS uncoupling in normotensive rat and SHR vessels.

To corroborate that cholesterol depletion in caveolae promotes eNOS uncoupling, we treated HUVECs with dextrin. The eNOS dimer/monomer ratio decreased (Fig. 8A,B). In addition, NO levels measured by DAF-2DA (Fig. 8E,F) were reduced, and ROS generation (Fig. 8G) measured by DHE augmented in HUVECs treated with dextrin, which confirmed eNOS uncoupling. This uncoupling occurs when either the substrate L-arginine or the redox active cofactor BH_4_ is not present^46,47^. Hence, pretreatment with BH_4_, L-arginine, or BH_4_ plus L-arginine avoided dextrin-induced eNOS monomerization in HUVECs (Fig. 8C,D). Moreover, these pretreatments boosted NO levels (Fig. 8E,F) and decreased ROS generation (Fig. 8G). The nonselective NOS inhibitor L-NAME also reduced the ROS levels induced by dextrin (Fig. 8G).

Taken together, our results confirm that caveolae structure and integrity are essential for endothelium-dependent relaxation. Additionally, a smaller number of caveolae is associated with hypertension. Finally, caveolae disruption promotes eNOS uncoupling in normotensive and hypertensive rat vessels and in HUVECs.

## Material and Methods

The Animal Care and Use Committees of the University of São Paulo (License CEUA 17.1.127.60.2) and the São Paulo State University (License CEUA 00423-2017) approved all the experimental protocols, which were conducted in accordance to the US National Institutes of Health (NIH Publication No. 85–23, revised 1996).

### Drugs

Phenylephrine (PE, L-phenylephrine hydrochloride), acetylcholine (ACh, acetylcholine chloride), methyl-β-cyclodextrin (dextrin), Nω-nitro-L-arginine methyl ester hydrochloride (L-NAME), dihydroethidium (DHE), calcium ionophore (A_23187_), 4,5-diaminofluorescein diacetate (DAF-2DA), N-(1-Naphthyl)ethylenediamine dihydrochloride, sulfanilamide, sodium nitrite and L-arginine were obtained from Sigma-Aldrich (St. Louis, MO – United States).

Coumarin-7-boronic acid (7-CBA) and tetrahydro-L-biopterin hydrochloride (BH_4_) were purchased from Cayman Chemical (Ann Arbor, MI – United States).

### Animals

Male SHRs (~300 g) aged 90 days and with systolic blood pressure ≥150 mmHg were used in the experiments. Age-matched male normotensive Wistar rats (~450 g) with systolic blood pressure ≤110 mmHg were employed as control. The systolic blood pressure was evaluated by tail plethysmography (PowerLab, ADInstruments, Melbourne, VIC – Australia). The animals were housed under standard laboratory conditions (at 22 ± 2 °C and under 12-h light/dark cycles) with free access to food and water.

### Vessel preparation

The Wistar (designated normotensive) rats and the SHRs were submitted to inhalation of isoflurane (BioChimico®, Itatiaia, RJ – Brazil), decapitated, and exsanguinated. Thoracic aortas and mesenteric artery beds were placed in two different Petri dishes containing cold Krebs-Henseleit solution [mM] (NaCl 130.00, NaHCO_3_ 14.9, C_6_H_12_O_6_ 5.5, KCl 4.7, KH_2_PO_4_ 1.18, MgSO_4_ 1.17, and CaCl_2_ 1.6; pH 7.4). The thoracic aortas and the mesenteric arteries were cleaned of connective tissue with the aid of a stereomicroscope (Luxeo 2S, Labomed, Los Angeles, CA – United States).

### Aorta ring vascular reactivity

The thoracic aortas were cut into 2-mm rings. To measure the isometric tension, the rings were placed between two stainless steel stirrups of a myograph (DMT, Melbourne, VIC – Australia). To develop basal active tension, the aortic rings were stretched until they reached 30 mN. Then, the rings were left under basal resting tension in oxygenated Krebs-Henseleit solution (95% O_2_ and 5% CO_2_; ph 7.4) at 37 °C for 60 min. Next, the rings were contracted with PE (1 µM), and the endothelial cell integrity was confirmed by over 90% and 70% relaxation in the aortas of Wistar rats and SHRs in response to ACh (10 µM), respectively. All the aortic rings used in this study presented intact endothelium.

### Resistance mesenteric artery ring vascular reactivity

The second or the third branch of the mesenteric arteries (internal diameter = 200–300 µm) was cut into 2-mm-long rings. The isometric tension in these rings was measured with a myograph (DMT, Melbourne, VIC – Australia). More specifically, two tungsten wires with diameter of 40 µm were placed through the lumen of the vessel, which was mounted according to the method described by Mulvany and Halpern^48^. The mesenteric artery rings were stretched to their optimal lumen diameter, to develop active tension. Then, the mesenteric artery rings were left under basal resting tension in oxygenated Krebs-Henseleit solution (95% O_2_ and 5% CO_2_; at 37 °C and pH 7.4) for 30 min. After that, the mesenteric artery rings were contracted with PE (1 µM), and over 80% and 60% relaxation in the arteries of Wistar rats and SHRs in response to ACh (10 µM), respectively, confirmed endothelial cell integrity. All mesenteric artery rings used in this study presented intact endothelium.

### Protocols

Cumulative concentration–response curves were constructed by using ACh and PE at concentrations ranging from 0.1 nM to 100 µM for the aortic rings and mesenteric artery rings of normotensive rats and SHRs in the absence of dextrin (control) or after incubation with dextrin (10 mM) for 60 min. To construct the concentration-response to ACh curves, the rings were contracted with PE (10 µM).

### Transmission electron microscopy

Procedures were performed as described by Moreira and collaborators^49^. Briefly, thoracic aortas and mesenteric artery rings treated with dextrin (10 mM) for 60 min or not (control) were fixed by immersion in a solution containing glutaraldehyde 2%, paraformaldehyde 2%, and sodium cacodylate buffer (0.1 M) for 12 h. After that, the preparations were kept in sodium cacodylate (0.1 M) at 4 °C until the next step was conducted. Post-fixation was carried out in osmium tetroxide 1% for 1 h. The samples were submitted to dehydration in a graded series of pure acetone. Then, the sample infiltration process was performed with a combination of Araldite resin and pure acetone (1:1 ratio overnight; 2:1 ratio for 4 h; 3:1 ratio for 4 h; and 100% resin for 24 h). Next, the material was polymerized with Araldite resin at 60 °C for 72 h. The plastic blocks were trimmed, and the 0.5-μm semi-thin sections (Leica ultra-microtome, Wetzlar, Germany) were stained with toluidine blue 1% to choose the appropriate areas for ultrathin sectioning (60–70 nm). Ultrathin sections were collected on Pyoloform and carbon-coated single-slot grids, and they were contrasted with uranyl acetate and lead citrate.

Electron micrographs were taken at an initial magnification of 10,000× and were photographically enlarged on the computer screen to a magnification of 50,000× with a transmission electron microscope (Hitachi H7500). Morphometry and quantitative analysis were performed with the ImageJ software from the National Institutes of Health. The caveolae were counted on the edge of the endothelial cell membrane. Results are expressed as the number of caveolae per square micrometer of the cell.

### Western Blot

The thoracic aortas and mesenteric arteries treated with dextrin (10 mM) for 60 min or not (control) were cleaned, frozen in liquid nitrogen, and stored at −80 °C. The samples were macerated in RIPA buffer supplemented with a cocktail of protease and phosphatase inhibitors. The homogenates were centrifuged at 4 °C and 12,000 rpm for 20 min, and the supernatants were collected for protein determination^50^. The total protein (10 μg) obtained from each sample was submitted to electrophoresis on polyacrylamide gel 12% and transferred to a nitrocellulose membrane. Then, the membrane was blocked with non-fat milk 5% for 1 h and incubated with primary antibodies against eNOS (1:1000, BD Bioscience, 610296), Cav-1 (1:2000, BD Bioscience, 610406), and p-eNOS Ser^1177^ (1:1000, Millipore, 07–428) at 4 °C overnight. Next, the membrane was incubated with secondary anti-rabbit (1:2000) or anti-mouse (1:2000) antibodies at room temperature for 60 min. The bands were detected by chemiluminescence with a radiographic film. GAPDH (1:1000, Santa Cruz, sc-365062) was used to normalize the results. The bands were quantified with the ImageJ Software (NIH Image).

### eNOS dimer/monomer low-temperature SDS-PAGE analyses

Human umbilical vein endothelial cells (HUVECs) were purchased from ATCC cell lines (American Type Culture Collection - Manassas, VA – United States) and cultured in growth medium (Dulbecco’s modified eagle’s medium – DMEM, Vitrocell, 00025) supplemented with fetal bovine serum (FBS) 10% from Gibco (12657029, South America) and used at passages 4–6. After serum deprivation in culture medium for 12 h, confluent HUVECs were treated with dextrin (10 mM) for 60 min or not (control) in the presence of BH_4_ (100 μM, for 30 min), L-arginine (1 mM, for 30 min), or BH_4_ plus L-arginine. Then, the cultured cells were lysed on ice in RIPA buffer supplemented with a cocktail of protease and phosphatase inhibitors, and the lysates were placed under non-denaturing conditions. The samples were prepared with 4x Laemmli sample buffer (Biorad, 1610747) plus betamercaptoethanol 10% (Biorad, 1610710) and were loaded on polyacrylamide gel 8%. During electrophoresis and protein transfer to the nitrocellulose membrane, the buffers were placed in an ice-water bath, and the whole apparatus was kept at 4 °C. The eNOS monomer and dimer forms were incubated with antibody against eNOS (1:1000, BD Bioscience, 610296) and α-tubulin or GAPDH were used to normalize the results. The bands were detected by a chemiluminescence substrate (Santa Cruz, SC-2048).

### Colorimetric Griess reaction

The thoracic aorta and mesenteric artery rings of normotensive rats and SHRs were incubated in a bath chamber containing Krebs-Henseleit solution (95% O_2_ and 5% CO_2_; pH 7.4) at 37 °C. The rings were treated with dextrin (10 mM) for 60 min or not (control). Some rings were incubated in the presence of L-NAME (1 mM, 30 min) or BH_4_ (100 µM, 30 min). Then, each sample was stimulated with PE (10 μM) followed by ACh (10 μM). Next, 50 μL of the bath solution of each sample was collected and added to 50 μL of Griess reagent (a 1:1 dilution of N-(1-Naphthyl)ethylenediamine dihydrochloride 1% in distilled water and sulfanilamide 1% in phosphoric acid 5%) in a 96-well plate. Sodium nitrite was used as standard. The standard curve ranged from 3 μM to 200 μM for nitrite. The absorbance was read at 540 nm. The results were normalized to total protein.

### Reactive oxygen species (ROS) production measurement with coumarin-7-boronate acid (7-CBA) or DHE probe

In a bath chamber, the thoracic aorta and mesenteric artery rings of normotensive rats and SHRs were incubated in 95% O_2_ and 5% CO_2_, pH 7.4, at 37 °C in the absence (control) or presence of dextrin (10 mM) for 60 min. Some rings were incubated with L-NAME (1 mM, 30 min) or BH_4_ (100 µM, 30 min). The rings were stimulated with PE (10 μM) followed by ACh (10 μM). Then, 50 μL of the bath solution of each sample was collected and added to 50 μL of 7-CBA (20 µM) for 30 min. The bath solution of each sample plus the probe was placed in a 96-well plate. The results were normalized to total protein.

The HUVECs were grown in a 96-well assay plate, washed twice with PBS (phosphate buffered saline), and incubated with medium containing DHE (2.5 μM) for 60 min. Then, the HUVECs were treated with dextrin (10 mM) for 60 min or not (control), which was followed by pretreatment with BH_4_ (100 μM, for 30 min), L-arginine (1 mM, for 30 min), L-NAME (100 μM, for 30 min), A_23187_ (5 μM, for 5 min), or BH_4_ plus L-arginine.

Fluorescence analysis of the oxidation product 7-OH-coumarin (COH, 332 nm/447 nm – Excitation/Emission)^51^ and the fluorescence produced by DHE (370 nm/420 nm – Excitation/Emission) were obtained on a SpectraMax M5 Microplate Reader (Molecular Advices, Sunnyvale, CA, United States).

### NO measurement with DAF-2DA probe

The HUVECs were grown in a 96-well assay plate, washed twice with PBS (phosphate buffered saline), and incubated with medium (DMEM, 0% FBS) containing DAF-2DA (2.5 μM) for 60 min. Then, the HUVECs were treated with dextrin (10 mM) for 60 min or not (control), which was followed by pretreatment with BH_4_ (100 μM, for 30 min), L-arginine (1 mM, for 30 min), L-NAME (1 mM, for 30 min) or BH_4_ plus L-arginine. The fluorescence produced by DAF-2DA was measured at 488 nm/530 nm (Excitation/Emission) on a SpectraMax M5 Microplate Reader (Molecular Advices, Sunnyvale, CA).

### Statistical analysis

For the functional studies, the EC_50_ (concentration of the agent that produced half-maximum amplitude) values were determined after logarithmic transformation of the normalized concentration–response curves and are reported as the negative logarithm (pD_2_). The maximum effect (ME) values are represented as percentage and are considered as the maximum amplitude response reached in the concentration−effect curves for the relaxation or contractile agents. The results are expressed as the mean ± standard error of the mean (SEM) of the obtained values; *n* indicates the number of animals, the number of thoracic aorta rings, or the number of mesenteric artery rings that were used in the experiments. In the experiments that employed HUVECs, *n* indicates independent experiments.

Two groups were compared by Student’s t-test; three or more groups were compared by one-way ANOVA with Tukey post-hoc test. Statistical analysis was performed with the Prism GraphPad 5.0 software. Differences were considered statistically significant when *p* < 0.05.
